# Amusements

**Published:** 1887-09

**Authors:** 


### HALL'S
### JOURNAL OF HEALTH

TRUTH DEMANDS NO SACRIFICE; ERROR CAN MAKE NONE.

VOL. 34. SEPTEMBER, 1887. No. 9.

### AMUSEMENTS.

There is nothing more conducive to health than innocent amusements, especially those which are coupled with exercise in the open air. In this country nearly all classes are so absorbed in money-making, that far too little time is given to recreation. Nature is in rebellion against such a one-sidedness of manly faculties, and shows her disapprobation in the thin-chested, stoop-shouldered, cadaverous forms that throng our public ways as they hurry hither and yon in their daily sacrifice of manly vigor for riches, *riches* which can have no value to the dying, and poorly compensate the wasted energies of an ill-spent life; ill-spent because both the mental and physical faculties were denied their needed sustenance. The English have fairly recovered from the puritanic palsy which largely overspread them in Cromwellian times. It was in those days that a voluminous author, writing in opposition to all manner of amusements, even the most innocent and juvenile, characterized the May festival and the flower dance around the May pole, as an invention of the devil, and a sure training for his sooty dominions, as if there could be any real affinity between brimstone and flowers. Within quite a short period, out-door games suited to both sexes, have become popular with us. The two leading ones of this description are croquet and lawn tennis, the latter being the present favorite. Match games of these, are frequent in our city parks, and suburban lawns. Nothing adds more to the picturesque, than the brilliantly costumed players in their good natured contention, which affords so much scope for the display of agility and grace. All have reason to rejoice that these healthy exercises, have become popularized, except perhaps, for purely business reasons, the family physician. But there is one feature in respect to these matters which is not so pleasing. No sooner has any exercise or game involving personal aptitude and skill gained a footing, than certain adepts seize the opportunity of exhibiting themselves for pay as champions, and these are not usually of a class conducive to its popularity. A few years ago roller skating rinks had a kind of epidemic run in England, owing mainly to the efforts of an American inventor. The amusement had its subsequent run in this country, and soon run out on account of the sportive tendencies of the *champions*, who turned them into spectacles for their benefit, and because too, of the want of proper management shown in their conduct, which made their patronage by the better classes, almost out of question. A season or two ago a female base ball club was organized and played for the edification of the public in different places, but we have heard nothing of it recently, and we opine it has been wisely abandoned. The exercise is too severe, and its perils too great for even the more robust of our sisters. But even among men this excellent game has met with a common fate, by being monopolized by teams of *professionals*, who vie with each other in their expert performances, which the eager public pay liberally to witness. Diversion is no longer the object. It is victory and gate money. An expert pitcher or catcher commands a larger salary than the principal of our common schools. It is unfortunate that nearly all our amusements are given over to this kind of competition and display, thus putting an end to the social neighborhood, or village games formerly so popular and health inspiring.

But fortunately the in door amusements are incapable of being absorbed in these ways. They belong more strictly to the family, and especially to the younger members, who make them a source of diversion at social gatherings. But one of the commonest sources of amusements, card playing, is frequently prohibited by the elders, not because there is any wrong in such a diversion, but out of fear that it may foster a love of gambling, a vice which cards are frequently made to serve. The same objection applies to various other things, even to wheat and flour and other necessaries, and we might add railroads and proprietory shares in almost every corporate enterprise. Yet we draw our dividends and eat our daily bread all the same. There is nothing quite so gloomy as a lugubrious male head of a family, unless it be the female head. They have a faculty of making life as nearly unendurable as is compatable with the ability to exist. Every one of our readers will be able to call to mind some one that fills this picture, and to experience a sense of gratitude that they live in a more wholesome atmosphere. The children of such parents, although ever so well cared for in other respects, are never happy and seldom robust. They cannot romp and play, it would be trivial, they cannot cheer the house with laughter, it would be indecorous. Such parents are ornamental only at funerals of the unconverted, where expectation has died out and hope is a thing of the past.

By all means make the home circle as cheerful and happy, as good nature and agreeable companionship can make it. The reward will be freedom from irrational restraint, good health and solid comfort.

The contrast of the two ways of directing the household, is just the difference between a prison and a home.

